# Silicon Hybrid EPDM Composite with High Thermal Protection Performance

**DOI:** 10.3390/polym16050695

**Published:** 2024-03-04

**Authors:** Chenyang Yan, Bo Chen, Xiangmei Li, Jiyu He, Xin Zhao, Yanli Zhu, Rongjie Yang

**Affiliations:** 1 National Engineering Research Center for Flame Retardant Materials, School of Materials Science and Engineering, Beijing Institute of Technology, Beijing 100081, China; 3120211151@bit.edu.cn (C.Y.); china17806240212@163.com (B.C.); yrj@bit.edu.cn (R.Y.); 2 Beijing Chemical Industry Research Institute, Co., Ltd., Beijing 100080, China; xm.lee@bit.edu.cn; 3 School of Mechatronical Engineering, Beijing Institute of Technology, Beijing 100081, China; zhuyanli1999@bit.edu.cn

**Keywords:** EPDM, silica, silica aerogel, thermal insulation

## Abstract

The effects of octaphenylsilsesquioxane (OPS), fumed silica, and silica aerogel on the thermal insulation properties of ethylene propylene diene monomer (EPDM) rubber were studied. On this basis, two kinds of fillers with good performances were selected to study the thermal insulation of an EPDM full-formula system. The results show that the addition of fumed silica or silica aerogel had a positive effect on the thermal insulation performance of EPDM rubber and its composite. A 30 wt% silica aerogel can be well dispersed in the EPDM rubber system and with a lower thermal conductivity compared with fumed silica. EPDM composite with 23.4 wt% fumed silica can produce more char residues at 1000 °C than at 500 °C in a burn-through test and formed the compact and porous char at 1000 °C, which had a lowest thermal conductivity. EPDM composite with fumed silica cannot be burned through 1000 °C burning, and comparison with silica aerogel revealed that it achieved the lowest back temperature and had a temperature of 388 °C after 800 s.

## 1. Introduction

Thermal protection material refers to a protective material added to the outer layer of the equipment when some equipment is working under harsh conditions, such as high temperatures outside, in order to protect the normal operation of the internal equipment and facilities [1,2]. EPDM composites have good thermal insulation performance, ablation resistance, and low density, which are widely used in thermal protection material. For example, EPDM composite is the most popular insulation material in the combustion chamber of solid rocket motors [3,4]. Moreover, EPDM rubber has good inclusiveness [5], and a large volume of research has addressed filler-reinforced EPDM insulation materials. Guo [6] added multi-walled carbon nanotubes (MWCNTs) to EPDM, which increased the carbon residue rate after thermal decomposition and improved the ablation performance of EPDM. Iqbal [7] found that a small amount of carbon nanotubes (CNTs) can significantly reduce the ablation rate of EPDM composites. In addition, as the number of CNTs increased, the backside temperature of the composites increased during the ablation test. Gao [8] used short poly(p-phenylene-2,6-benzobisoxazole) (PBO) fibers to reinforce EPDM as thermal insulation materials, and the mass loss rate and erosion rate of the PBO fibers/composites were 0.05 g s^−1^ and 0.10 mm s^−1^.

Silica powder is widely used to improve the char retention and thermal protection performance. Wollastonite [9] or nano-sized silica [10] is added to increase the heat of ablation, improve the char retention, and decrease the oxidation rate of the charred material. A blend of EPDM/polyimide rubber, compounded with nano-silica, exhibited a desirable improvement in thermal conductivity as well as thermal stability of the corresponding composites [11]. The blending of the micro- and nano-sized SiO_2_ additives in EPDM and silicone rubber resulted in higher thermal stability [12,13]. Jiang [4] studied the effect of OPS on the fire and ablation performances of EPDM composites and found that OPS lowered the linear ablation rate of these composites and improved their flame retardancy.

The thermal protection performance needs to be evaluated in extremely severe thermal flux environments during the re-entry stage, missile launching systems, and the solid rocket motors of space vehicles, and ablative rate by oxyacetylene flame testing is a key performance metric [4]. Silica aerogel has nano-scale particles, pore size, low density, large open pores, high specific surface area, and low thermal conductivity [14]. Aerogel, includes oxide aerogels, carbon aerogels, and organic aerogels, and thermal barrier coatings were applied to aerospace thermal protection; thermal conductivity is an important property [15,16]. Additionally, the thermal runaway of a lithium ion battery in an extreme fire condition required protection, and the burn-through test is a necessary test for investigating the insulation of materials [17].

There remains a lack of knowledge and understanding of the effects of different silicon hybrid filler systems on EPDM rubber thermal protection properties. Especially, it is necessary to better understand the burn-through behavior of thermal protection materials. In this paper, the morphology, burn-through properties, and thermal properties of silica, OPS, and silica aerogel were investigated in EPDM rubber and EPDM composites. The thermal protection performance and mechanism will be discussed.

## 2. Experimental Section

### 2.1. Materials

EPDM, LANXESS; sulfur (S), Luoyang Sanrui Industrial Co., Ltd., Luoyang, China; liquid paraffin wax (LPW), Guangdong Shantou Xilong chemical plant, Shantou, China; N-cyclohexyl-2-benzothiazolisulfonamide (accelerator, CZ), Tianjin Kemai Chemical Co., Ltd., Tianjin, China; 1,3-diphenylguanidine (accelerator, D), Beijing Chemical Reagent Company, Beijing, China; Di-(tert-butyl isopropyl peroxide) benzene (curing agent, BIPB), Shanghai Sundi Chemical Co., Ltd., Shanghai, China; silane coupling agent (coupling agent, Si-69), Shandong Evonik Lanxing Chemical Industry Co., Ltd., Rizhao, China; fumed silica (particle size: 45 μm), Beijing Howell Chemical Products Limited Liability Company, Beijing, China; silica aerogels (particle size: 18 μm), IBIH Advanced Material Co., Ltd., Huanghua, China; octaphenylsilsesquioxane (OPS) (particle size: 20–50 μm), BIT Flame Retardant Science and Technology Ltd., Beijing, China; stearic acid (plasticizer), Beijing Chemical Reagent Company, Beijing, China; zinc borate (activator, ZnB), Beijing Tongguang Fine Chemical Company, Beijing, China; aramid pulp, Beijing Howell Chemical Products Limited Liability Company, Beijing, China; aluminum diethylphosphinate (ADP), Beijing Tongguang Fine Chemical Company, Beijing, China; phenolic resin, Beijing Chemical Reagent Company, Beijing, China.

### 2.2. Preparation of Samples

#### 2.2.1. Preparation of Silicon Hybrid EPDM

Firstly, silicon hybrid EPDM was mixed on a double-roll mixer (Model: LRMR-S-50/W, Labtech Engineering Co., Ltd., Berlin, Germany.) for 10–20 min; liquid paraffin wax, Si-69, silicon filler (OPS, fumed silica, silica aerogel), accelerator CZ, accelerator DCP, and curing agent BIPB were added sequentially after a period of time and were fully mixed. Secondly, the respective blends were transferred to specific molds with 100 mm × 100 mm × 3 mm thicknesses. The final silicon hybrid EPDM was obtained by curing for 45 min in a compression press (Model: P 300 P/M, COLLIN LAB & PILOT SOLUTIONS, Berlin, Germany.) at 160 °C and 15 MPa. An EPDM-0 sample without silicon filler was prepared under the same curing conditions as a control sample. The formulation of the silicon hybrid EPDM is shown in Table 1 below.

#### 2.2.2. Preparation of Silicon Hybrid EPDM Composites

Silicon hybrid EPDM composites were mixed on a double-roll mixer (Model: LRMR-S-50/W, Labtech Engineering Co., Ltd.) for 10–20 min. EPDM-00 is a control sample of EPDM composites and was prepared by additionally adding aramid pulp, aluminum diethylphosphinate (ADP), phenolic resin, and ZnB on the base of EPDM-0. After thorough mixing by the effect of shear action, the respective blends were transferred to specific molds with 100 mm × 100 mm × 3 mm thicknesses. The final silicon hybrid EPDM composites were obtained by curing for 45 min in a compression press (Model: P 300 P/M, COLLIN LAB & PILOT SOLUTIONS) at 160 °C and 15 MPa. The formulation of silicon hybrid EPDM composites is shown in Table 2 below. After analyzing the dispersion, the mechanical properties and thermal insulation properties of the four groups of samples are shown in Table 1; the dispersion and thermal insulation properties of EPDM-OPS were worse than those of EPDM-Si and EPDM-Gel, so only the remaining three groups of samples were left in Table 2 to prepare composite materials for further performance comparison.

### 2.3. Characterization

#### 2.3.1. Thermogravimetric Analysis (TGA)

TGA was performed over the range of 40–900 °C on a NETZSCH 209 F1 thermal analyzer(Berlin, Germany) at a heating rate of 10 °C·min^−1^ under a nitrogen atmosphere at a flow rate of 100 mL·min^−1^. The mass range of the samples was 2–5 mg. Samples were examined in duplicate, and average values are reported.

#### 2.3.2. Burn-Through Test

**Figure 1:**
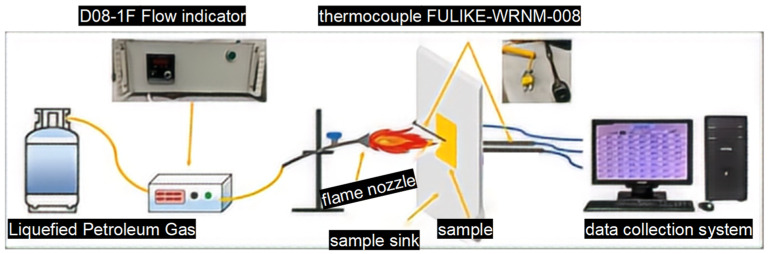
Flame combustion high-temperature test system schematic diagram.

A burn-through test was used to evaluate the thermal insulation performance of silicon hybrid EPDM rubber and composites. The burn-through test system was created by our lab. Liquefied petroleum gas (LPG) was used as the ignition source in this setup. Gas flow was regulated using a D08-1F flow display instrument (Qixinghuachang, Shenzhen, China), and the test temperature was adjusted by changing both gas flow and the horizontal distance between the flame nozzle and the front of the sample. The flame nozzle being 18.6 cm from the sample with a gas flow rate of 6 L/min could obtain a 500 °C flame. At the same time, the flame nozzle being 7.7 cm from the sample with a gas flow rate of 6 L/min could obtain a 1000 °C flame, as shown in Figure 1.

#### 2.3.3. Thermal Conductivity

Thermal conductivity was obtained using an LFA 467 flash thermal conductivity meter by NETZACH (Berlin, Germany). The thermal diffusivity and specific heat of the silicon hybrid EPDM rubber and char residues after the silicon hybrid EPDM composite burned and the thermal conductivity of EPDM rubber and its char were calculated by thermal diffusivity and specific heat; the sample size was a 25 mm × 25 mm × 3 mm block sample or char powder, and the temperature was set at 25 °C.

#### 2.3.4. Field-Emission Scanning Electron Microscope (FSEM)

An ultra-high-resolution field-emission scanning electron microscope created by Hitachi, Tokyo, Japan, FSEM (SU8020), analyzed the micromorphology of the silicon hybrid EPDM and silicon hybrid EPDM composite char after heating in the cone calorimeter close to 1000 °C. The dispersion of the silicon filler in EPDM and the char structure of EPDM composites was recorded.

#### 2.3.5. High-Performance Fully Automatic Mercury Injection

The pore surface area, pore volume, porosity, average pore diameter, pore distribution curve, apparent density, and true density of the char skeleton of silicon hybrid EPDM composites can be obtained by using the AutoPore V9620 high-performance automatic mercury injection instrument produced by McMuriatic Co., Ltd., New York, NY, USA. The char was obtained by a thermal insulation performance test at 1000 °C and was ground into powder for testing.

#### 2.3.6. Scanning Electron Microscopy (SEM)

Scanning electron microscopy (SEM) images were obtained with an FEI Quanta 650, New York, NY, USA(FE-SEM), and the acceleration voltage was 10 kV.

#### 2.3.7. Mechanical Properties

The mechanical properties were tested according to the GB/T 528-2009 standard [18] by using an MTS CMT4104 electronic universal testing machine (MTS System Co. Ltd., Beijing, China). The EPDM sample was cut into the corresponding dumbbell-shaped sample No. 2 using a 2-type cutter, with no less than 5 samples in each group. The tensile rate was set to 500 mm/min, and the test ambient temperature was 20 °C.

#### 2.3.8. X-ray Photoelectron Spectroscopy (XPS)

X-ray photoelectron spectroscopy (XPS) results were obtained with the PHI Quantera-II SXM (ULVAC-PHI, Tokyo, Japan) of the Beijing Institute of Technology Advanced Materials Experiment Center. The element content information on the surface of the sample was obtained under a vacuum of lower than 10^−6^ Pa.

#### 2.3.9. TG-FTIR

A TG (Netzsch 209 F1,Berlin, Germany) and Nicolet 6700 FT-IR spectrometer were connected by a capillary tube. The temperature range was 40–700 °C, and the heating rate was 20 K/min. The thermogravimetric product entered the infrared gas detection pool through the capillary tube.

## 3. Results and Discussion

### 3.1. Morphology of Fillers in Silicon Hybrid EPDM

**Figure 2:**
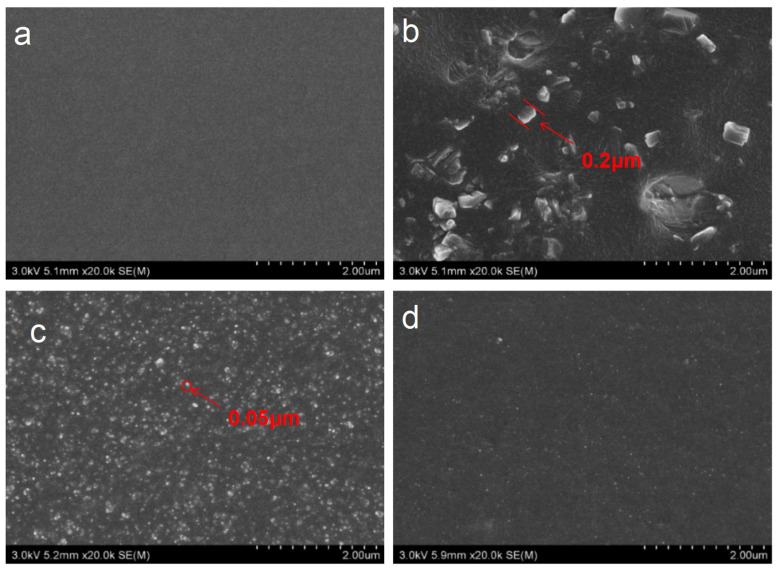
SEM images of silicon fillers in EPDM: (**a**) EPDM-0, (**b**) EPDM-OPS, (**c**) EPDM-Si, and (**d**) EPDM-Gel.

The SEM micrographs of different silicon fillers in EPDM are shown in Figure 2. OPS is a crystalline structure that is easier to aggregate than fumed silica and silica aerogel, and the SEM of EPDM-OPS showed some aggregates ranging 10–15 μm, indicating inadequate filler dispersion within the EPDM. Meanwhile, the fumed silica particles are homogeneously dispersed in the EPDM matrix, and adding silica aerogel into EPDM led to a better homogenous morphology with well-dispersed and distributed inclusions. The particles of aerogel can be hardly seen in the EPDM. Silica aerogel is more evenly distributed than fumed silica. The extremely high porosity, high specific surface area, and high pore volume make the silica aerogels less likely to aggregate and more likely to disperse into individual particles during mixing in the double-roll mixer. In contrast, the pore volume of fumed silica is small, and the specific surface area is only one-third of that of silica aerogel [19].

**Figure 3:**
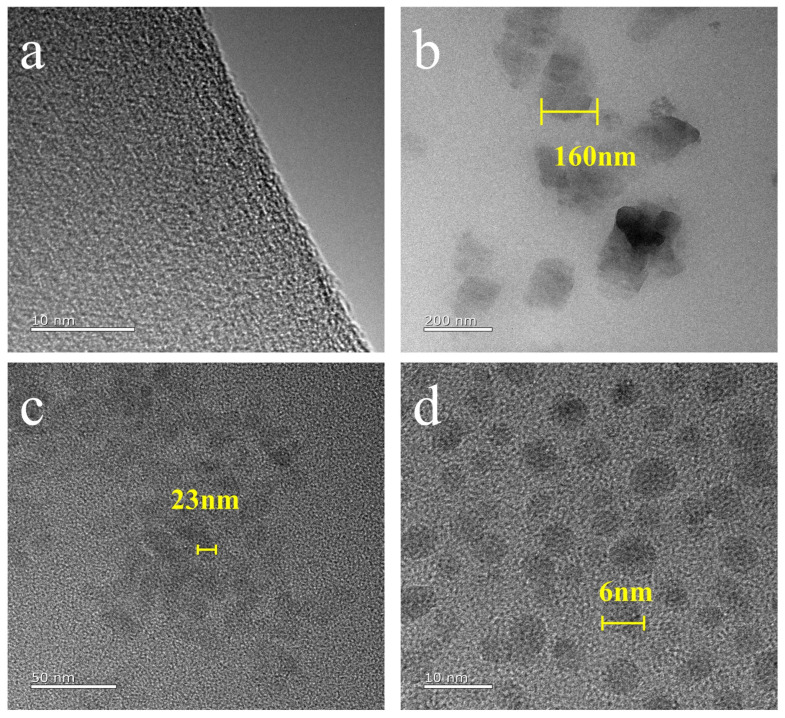
TEM images of silicon fillers in EPDM: (**a**) EPDM-0, (**b**) EPDM-OPS, (**c**) EPDM-Si, and (**d**) EPDM-Gel.

In order to further explore the microscopic dispersion state of OPS, fumed silica, and silica aerogel in EPDM, we used TEM to perform our observation. As shown in Figure 3c,d, most fumed silica and silica aerogel can be dispersed in the matrix during the 3–25 nm size. As can be seen from Figure 3b, most of the OPS is easy to aggregate into irregular blocks ranging 160–200 nm, indicating that OPS is more inclined to aggregate into nanoparticles in the resin.

It can be seen from Table 3 that silica and aerogel have the most significant improvement on the tensile strength and elongation at the break of EPDM. Silica and aerogel are excellent reinforcing agents for rubber. The active hydroxyl groups on the surface of silica and aerogel particles form hydrogen bonds with the hydrogen on the organic macromolecular chain. The interaction between particles, the ‘bridge’ chain between silica (aerogel) –polymer–silica (aerogel) and silica (aerogel) aggregates, constitutes a spatial network structure. These interactions can play a reinforcing role in the mechanical properties of EPDM. Secondly, the specific surface area of the aerogel used in the experiment is 621 m^2^/g, the total pore volume is 3.09 mL/g, the specific surface area of silica is 190 m^2^/g, the total pore volume is 0.64 mL/g, the specific surface area of the aerogel is 3.3 times that of silica, and the pore volume is 4.8 times that of silica. From the TEM of Figure 3, it can be seen that the particle size of the aerogel is smaller and the dispersion in EPDM is better. Therefore, compared with silica, the reinforcing effect of aerogel on EPDM is better.

### 3.2. Thermal Degradation and Insulation of Silicon Hybrid EPDM

**Figure 4:**
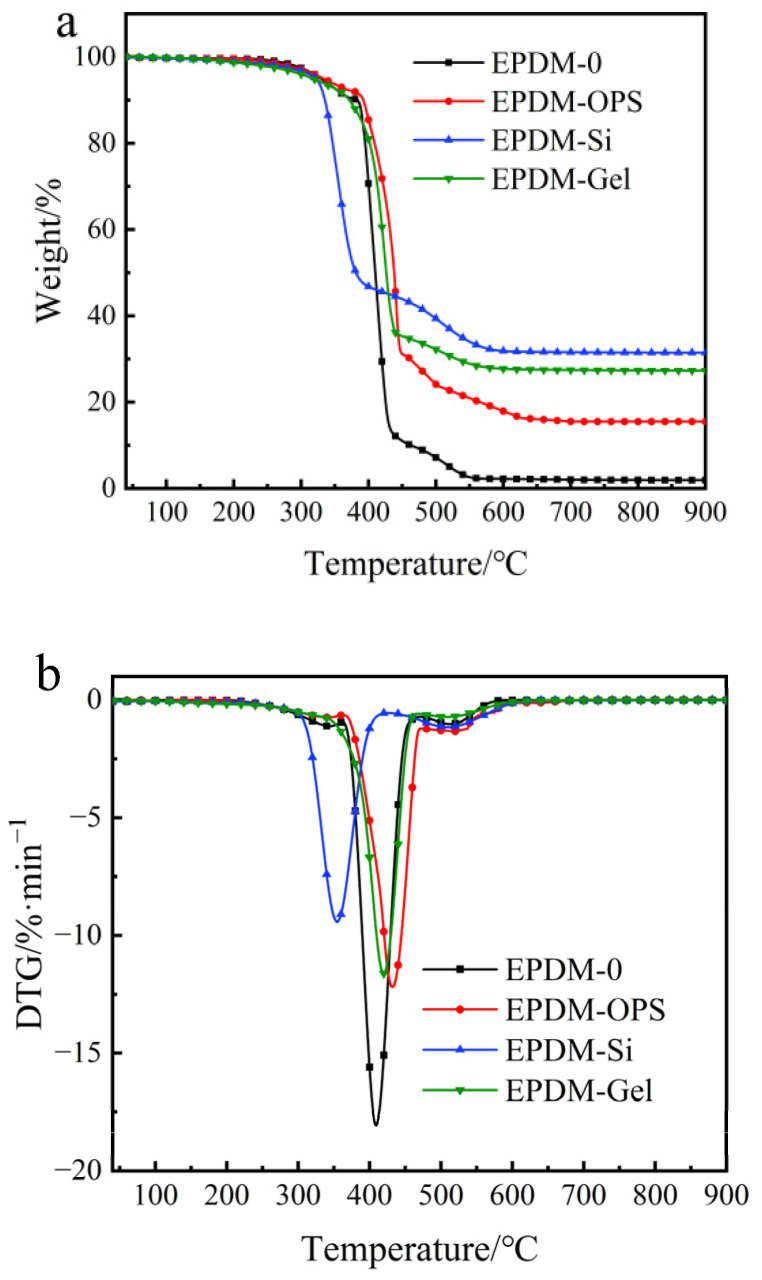
TGA–DTG curve of silicon hybrid EPDM in air. (**a**) TGA curve (**b**) DTG curve.

The TGA curves of the EPDM and silicon fillers in EPDM are shown in Figure 4 and Table 4. Compared with the amount of char in EPDM-Si and EPDM-Gel, the presence of OPS in the vulcanized rubber had lower carbon residue content.

Theoretical Value Char Residue can be calculated in the TGA-DTG curves of different fillers in air atmosphere in the Supplementary Materials.

**Figure 5:**
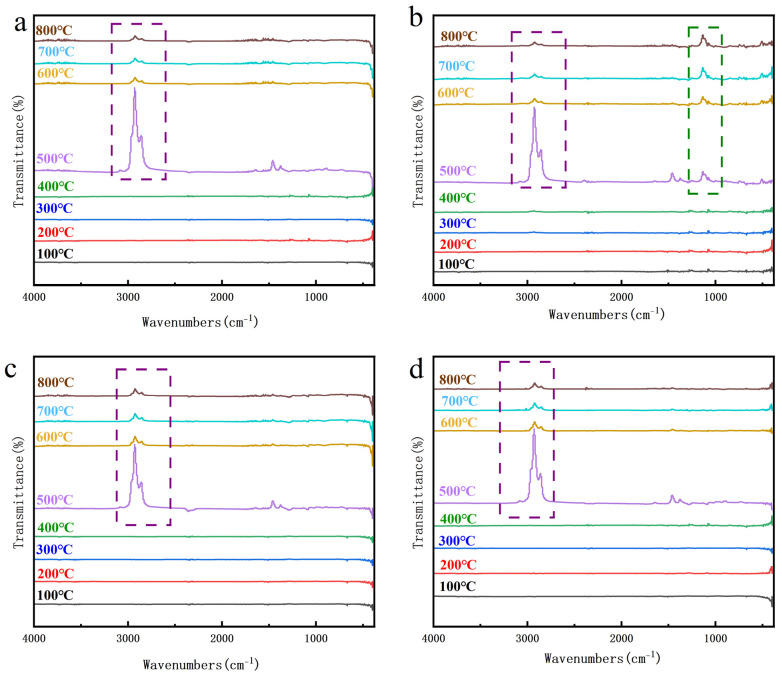
TG-FTIR curve of silicon hybrid EPDM in air. (**a**) TG-FTIR curve of EPDM-0 (**b**) TG-FTIR curve of EPDM-OPS (**c**) TG-FTIR curve of EPDM-Si (**d**) TG-FTIR curve of EPDM-Gel purple box: Infrared absorption peak at 2980 cm^−1^ green box: Infrared absorption peak at 1140cm^−1^ The black line represents the TG-FTIR curve at 100 °C, the red line represents the TG-FTIR curve at 200 °C, the blue line represents the TG-FTIR curve at 300 °C, the green line represents the TG-FTIR curve at 400 °C, the purple line represents the TG-FTIR curve at 500 °C, the yellow line represents the TG-FTIR curve at 600 °C, the light blue line represents the TG-FTIR curve at 700 °C, the brown line represents the TG-FTIR curve at 800 °C.

It can be seen from the infrared spectra (Figure 5) of EPDM-OPS at different temperatures that the infrared absorption peak appeared at 1100 cm^−1^ at 500 °C, and the Si–O bond [20] was broken during the decomposition process so that the substances involved in charring were reduced during the charring process. It can also be concluded from Table 5 that the Si content of EPDM-OPS was significantly lower than that of EPDM-Si and EPDM-Gel. The content of O and Si in EPDM-Si and EPDM-Gel increases, and more silicon–oxygen network structures are formed in the residual carbon, which improves the stability of the carbon layer so that the carbon layer can greatly hinder the heat transfer, thereby improving the thermal insulation performance [21].

**Figure 6:**
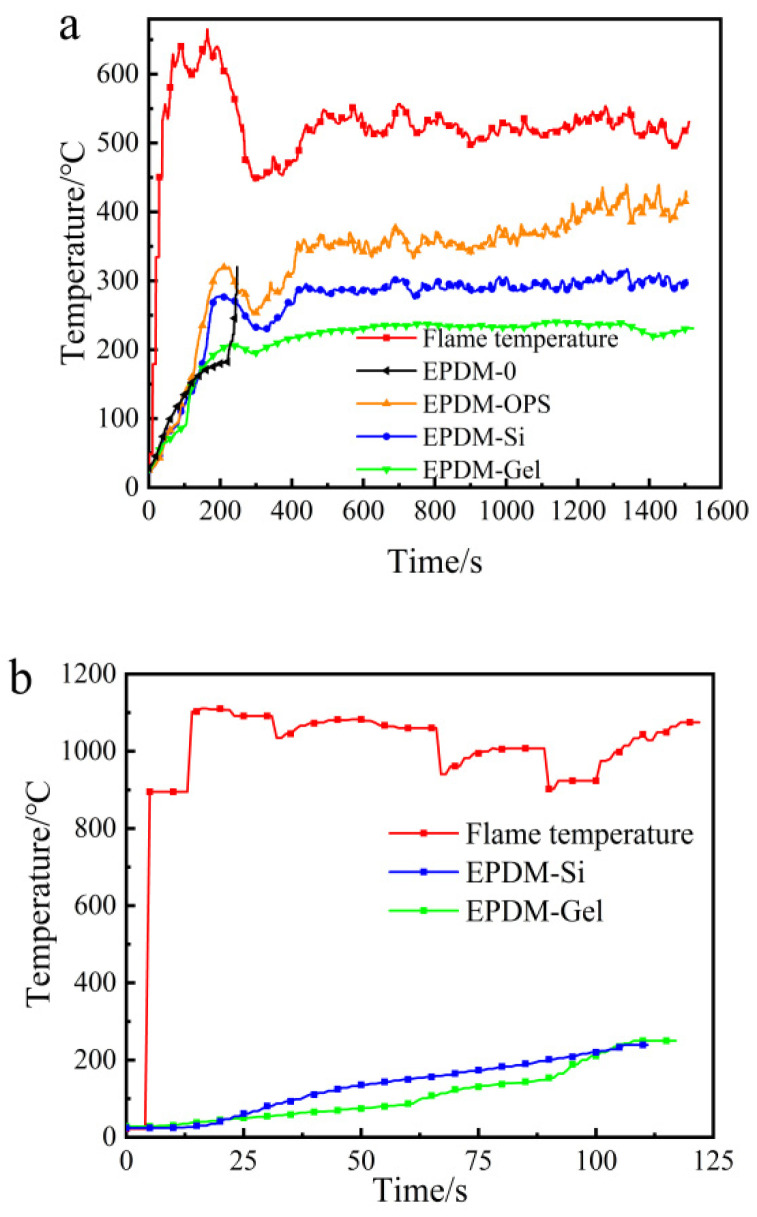
The back temperature curve of silicon hybrid EPDM at (**a**) 500 °C and (**b**) 1000 °C flame.

**Figure 7:**
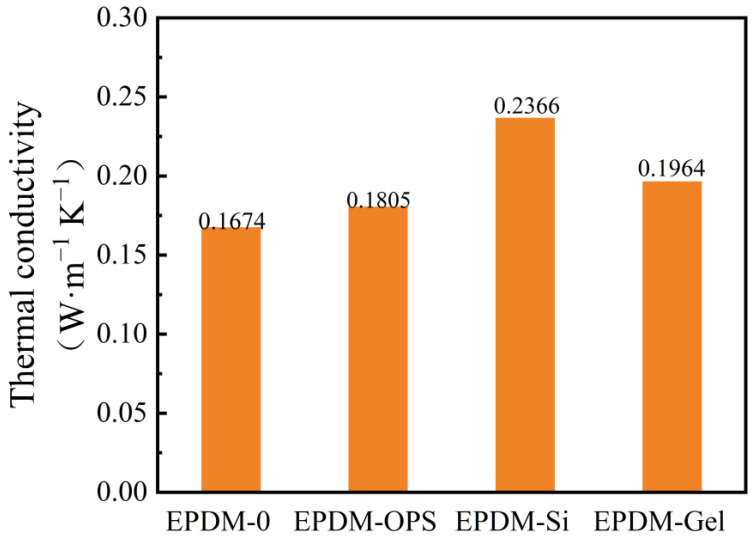
Thermal conductivity of silicon hybrid EPDM.

In order to investigate the burn-through behavior of OPS, fumed silica, and silica aerogel on EPDM at 500 °C or 1000 °C, we performed testing using a flame combustion high-temperature test system. It can be seen from Figure 6a that when the flame torch at 500 °C acted on the EPDM-OPS system, it could not prevent the flame from continuously destroying the matrix, and EPDM-OPS would be burned through. Fumed silica and silica aerogel dramatically increased the char residue of EPDM, and the generated char layer can effectively protect the matrix and avoid the continuous ablation by flame. The back temperature curve of EPDM-Gel is lower than that of EPDM-Si within 1500 s of ablation. Based on the poor thermal insulation performance of EPDM-OPS and the weak ability to resist high-temperature flame air flow erosion for a long time, Figure 6b only showed the thermal insulation performance of EPDM-Si and EPDM-Gel for a 1000 °C flame. It can be seen that EPDM-Si and EPDM-Gel are burned through when the back temperature reaches 250 °C, and the time to resist the flame was maintained for 110 s from Figure 6b and Table 6. EPDM-Gel has a good thermal insulation performance compared with EPDM-Si. On the one hand, this benefits from the large specific surface area, small pore size, and good dispersion of silica aerogel from Figure 2. On the other hand, the low thermal conductivity of EPDM-Gel is an important reason leading to the low back temperature in Figure 7.

### 3.3. Thermal Degradation and Insulation of Silicon Hybrid EPDM Composites

**Figure 8:**
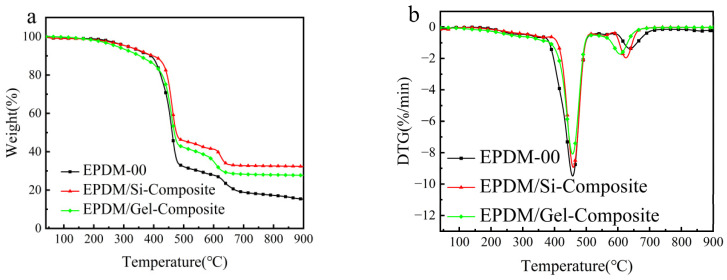
TG and DTG curves of silicon hybrid EPDM composites in air. (**a**) TG curves (**b**) DTG curves.

The TG (weight loss curves) and DTG (derivative weight loss) patterns of EPDM composite thermal protection material are shown in Figure 8. Silicon hybrid EPDM composites show obvious two-step decomposition characteristics. The first stage of thermogravimetric decomposition mainly occurs between 400 °C and 500 °C, and this stage is mainly the thermal decomposition of EPDM, fumed silica, and silica aerogel fillers. The second stage of thermal degradation mainly occurs between 550 °C and 650 °C, and this stage is mainly the thermal decomposition of EPDM, phenolic resin, and reinforcing agent.

From Table 7, it can be seen that the char residue of EPDM/Si-Composite material is the highest. Compared with EPDM-00, EPDM/Gel-Composite significantly reduces the initial decomposition temperature (T_5%_), which may be due to the mass loss caused by the volatilization of residual low-boiling organic matter inside the silica aerogel or the decomposition or oxidation of hydroxyl, methyl, and other organic groups on the surface, which makes the growth time of char layer of EPDM/Gel-Composite longer, which further results in a denser char layer.

**Figure 9:**
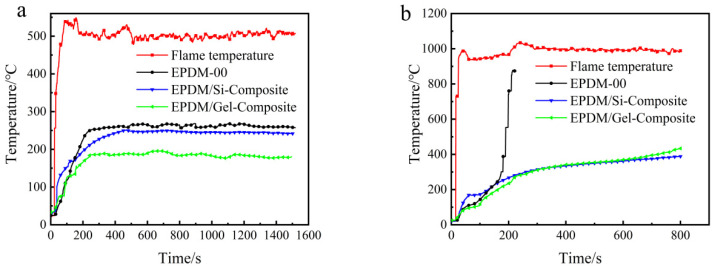
Back temperature curve of silicon hybrid EPDM composites: (**a**) 500 °C flame, (**b**) 1000 °C flame.

**Figure 10:**
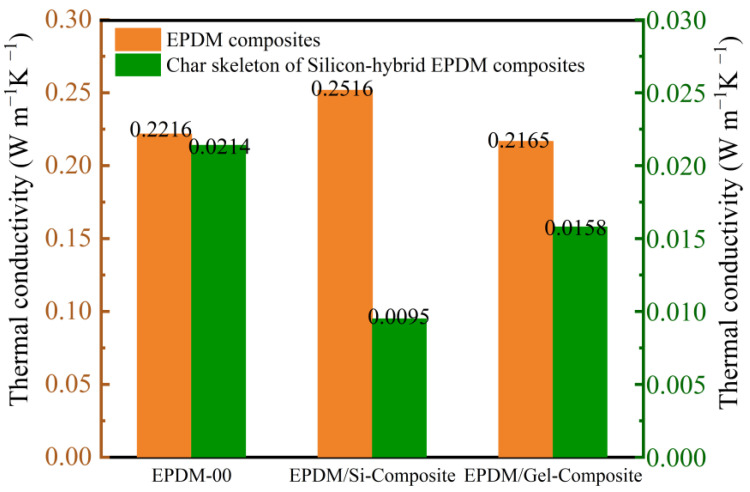
Thermal conductivity of the char of silicon hybrid EPDM composites.

We further investigated the burn-through behavior of silicon hybrid EPDM composites at a 500 °C or 1000 °C flame torch. Based on the poor thermal insulation performance of EPDM-OPS in the previous section, we only investigated the thermal insulation performance of EPDM/Gel-Composite and EPDM/Si-Composite. It can be seen from Figure 9a and Table 8 that when the flame torch at 500 °C acted on the silicon hybrid EPDM composites system, the back temperature of EPDM/Gel-Composite was 176–186 °C. It was lower than EPDM/Si-Composite, which was 242–250 °C. However, in Figure 9b, the back temperature curve of EPDM/Si-Composite is always higher than EPDM/Gel-Composite. The back temperature of EPDM/Gel-Composite is higher than EPDM/Si-Composite for the 1000 °C flame as time increases in Table 8. In the insulation performance test for the 1000 °C flame torch, the back temperature curves of EPDM/Si-Composite, EPDM-Composite, and EPDM/Gel-Composite conform to the law of thermal conductivity of silicon hybrid EPDM composites, according to Figure 9. With the extension of ablation time and the increase in the char layer, the thermal conductivity of silica aerogel char skeleton is larger than that of the fumed silica char skeleton from Figure 10. Therefore, EPDM/Gel-Composite has a significant growth rate of heating up, while the heating trend of EPDM/Si-Composite remains stable. At the 500 °C flame, the thermal protection performance of silicon hybrid EPDM composites is affected by the thermal conductivity of the silicon hybrid EPDM composites matrix. Meanwhile, at the 1000 °C flame, the thermal protection performance of silicon hybrid EPDM composites is affected by the thermal conductivity of the carbon skeleton of silicon hybrid EPDM composites.

### 3.4. Char of Silicon Hybrid EPDM Composites

**Figure 11:**
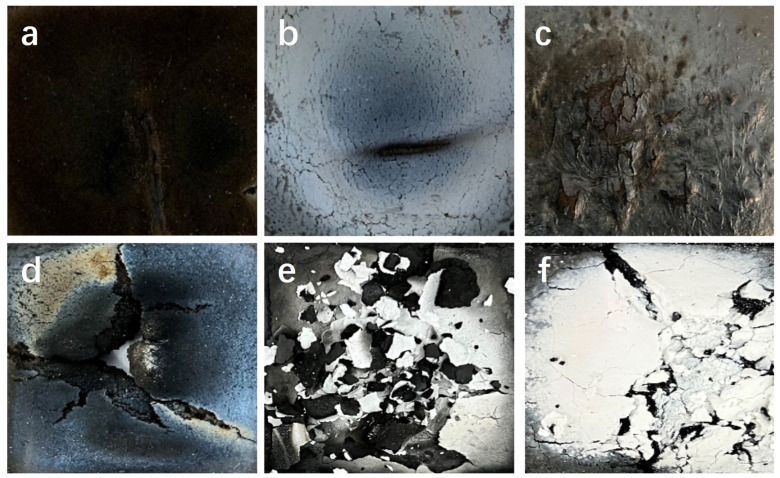
The front photo of fire ablation of silicon hybrid EPDM composites exposed to a 500 °C (**a**–**c**) for 1600 s and 1000 °C (**d**–**f**) flame for 800 s. (**a**,**d**) EPDM-00, (**b**,**e**) EPDM/Gel-Composite, and (**c**,**f**) EPDM/Si-Composite.

From Figure 11, it can be seen that the char of silicon hybrid EPDM composites increases but causes serious damage on the surface at 1000 °C compared with 500 °C. The char of EPDM/Si-Composite is denser than EPDM/Gel-Composite at 1000 °C, and this char morphology is exactly the opposite of 500 °C. As seen in Figure 11a, the back temperature curve of EPDM/Si-Composite is always higher than EPDM/Gel-Composite at 500 °C. It is worth noting that silicon hybrid EPDM composites are not decomposed completely under the flame ablation at 500 °C for 1600 s. The content of char generated by the EPDM thermal protection material is relatively small, and the undecomposed layer still accounts for the main part. The back temperature depends more on the thermal conductivity of the silicon hybrid EPDM composites matrix.

**Figure 12:**
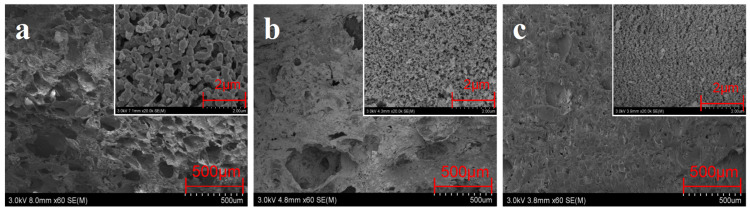
FSEM photo of the inner char layer of silicon hybrid EPDM composites at 60×, 20.0 k×. (**a**) EPDM-00, (**b**) EPDM/Gel-Composite, and (**c**) EPDM/Si-Composite.

The closely and firmly packed char is an important reason to reduce the back temperature of EPDM/Si-Composite. In order to further study the microstructure of the char of silicon hybrid EPDM composites, the char was obtained by a cone calorimeter test at close to 1000 °C, and the inner char was used for observation by FSEM. From the scanning electron microscope diagram in Figure 12, it can be seen from the morphology of 20.0 k that the char skeleton of EPDM-00 is thick and has obvious boundaries; the pore diameter between the skeletons is large and unevenly dispersed. The addition of fumed silica and silica aerogel makes the char skeleton become finer and denser, and the pore size is small and uniformly dispersed. Especially, the inner char of EPDM/Si-Composite is more compact than EPDM/Gel-Composite. Moreover, the thermal conductivity of the char skeleton of EPDM/Si-Composite is low; compared with EPDM/Gel-Composite and EPDM-00, the thermal conductivity of fumed silica and silica aerogel char skeleton is reduced by 89–97%. Therefore, the thermal conductivity of the char layer plays a major role in heat insulation under a 1000 °C flame.

### 3.5. Thermal Insulation Mechanism

The char of silicon hybrid EPDM composites was prepared to use the char after the thermal protection performance testing at 1000 °C. To analyze the pore parameters of char, char was ground into powder. The pore volume, total pore area, average pore diameter, and porosity are listed in Table 9. The addition of fillers reduces the porosity and pore size of the char layer from Table 9. Compared with EPDM/Si-Composite, the pore size and pore volume of EPDM/Gel-Composite’s char skeleton are smaller, which also forms a denser char layer. As can be seen from Figure 11, after flame ablation at 1000 °C, the surface of the char layer of EPDM/Gel-Composite is seriously damaged. The char layer derived from the aerogel presents a significant pore size, whereas the char skeleton exhibits smaller and fewer pores, accompanied by a thin carbon skeleton. In contrast, the char skeleton formed by EPDM/Si-Composite is thick, enabling the formation of a complete char layer, and the skeleton shows greater porosity and pore dimensions.

From the scanning electron microscope photo (Figure 2), it can be seen that fumed silica and silica aerogel are well dispersed in EPDM, which cause the thermal stability of both to be improved; the layer’s extended growth time increases residue char, aiding in uniform layer formation.

At 500 °C, the char layer is proportionally small, forming an ultra-thin char layer, as the matrix material undergoes significant expansion. Porosity’s influence is limited in this context, with the primary factor being the matrix’s thermal conductivity. EPDM/Gel-Composite’s low thermal conductivity contributes to its strong performance in the 500 °C ablation test. At 1000 °C, the char layer’s proportion experiences a substantial rise, leading to increased porosity and lowered char layer thermal conductivity. Consequently, the char layer assumes a pivotal role. The micrometer holes in the char layer of EPDM/Si-Composite are large, which makes the char layer have low thermal conductivity. The presence of a complete char layer and the low thermal conductivity of EPDM/Si-Composite char layer contribute to its impressive performance at 1000 °C.

**Figure 13:**
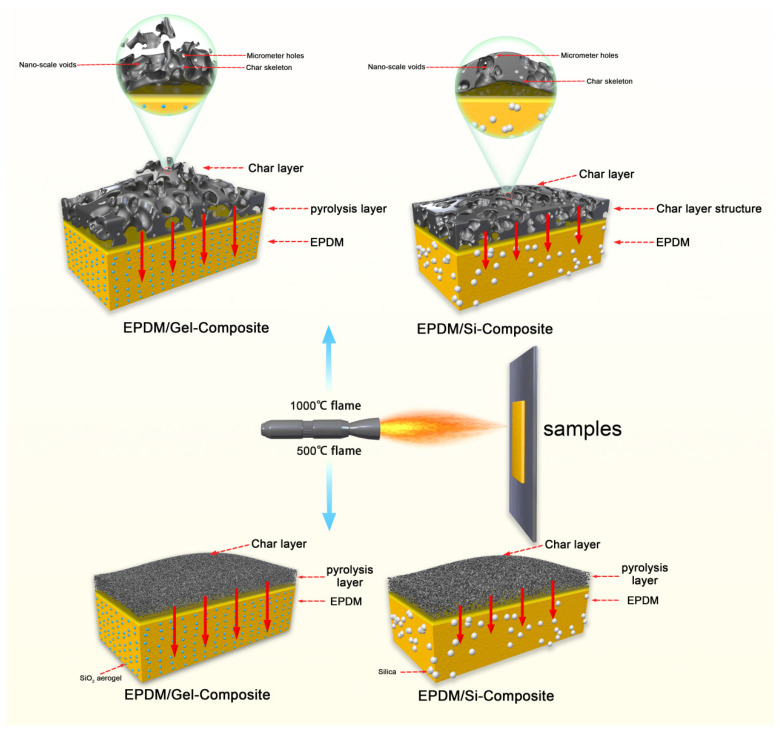
Schematic diagram of the principle of thermal insulation of silicon hybrid EPDM composites.

Moreover, it can be seen that at 500 °C, the proportion of char is relatively small, consisting of only a very thin char layer, and the matrix material expands greatly. At this time, the influence of porosity is small, mainly based on the thermal conductivity of the matrix. At 1000 °C, the proportion of the layer increases significantly, the porosity of layer is large, and the thermal conductivity of the overall composites is further reduced. At this time, the layer plays a main protective role. The insulation schematic diagram of a silicon hybrid EPDM composite is shown in Figure 13.

## 4. Conclusions

The addition of fumed silica or silica aerogel can obviously improve the thermal insulation performance of silicon hybrid EPDM thermal protection composites under 500 °C or 1000 °C flames. EPDM/Gel-Composite had the best thermal insulation performance under the flame of 500 °C, and the back temperature was stable at 176–186 °C. EPDM/Si-Composite showed the best thermal insulation performance under the flame of 1000 °C, and the back temperature was only 388 °C and lasted for 800 s.

It was found that the thermal conductivity of silicon hybrid EPDM thermal protection composite matrixes mainly affects the thermal insulation performance under a 500 °C flame, while the thermal conductivity of the char skeleton mainly affects the thermal insulation performance under a 1000 °C flame.

The char of EPDM thermal protection composites is a kind of porous layer, and the porosity of char is above 60%. The pore diameter of the char skeleton in the addition of fumed silica is larger than the added silica aerogel, leading to the char skeleton being thick. Meanwhile, the char skeleton is also very compact between them compared with the EPDM composite adding silica aerogel.

## Figures and Tables

**Table 1:** The formulation of the silicon hybrid EPDM.

Component	EPDM (wt%)	OPS (wt%)	Fumed Silica (wt%)	Silica Aerogel (wt%)	LPW (wt%)	CZ (wt%)	D (wt%)	Si-69 (wt%)	BIPB (wt%)
EPDM-0	90	/	/	/	2.4	1.2	0.3	3	3.1
EPDM-OPS	60	30	/	/	2.4	1.2	0.3	3	3.1
EPDM-Si	60	/	30	/	2.4	1.2	0.3	3	3.1
EPDM-Gel	60	/	/	30	2.4	1.2	0.3	3	3.1

**Table 2:** The formulation of the silicon hybrid EPDM composites.

Composite	EPDM-0 (wt%)	Fumed Silica (wt%)	Silica Aerogel (wt%)	Fiber Pulp (wt%)	ZB (wt%)	ADP (wt%)	Phenolic Resin (wt%)
EPDM-00	61.3	0	0	4.9	6.1	15.3	12.4
EPDM/Si-Composite	46.9	23.4	0	3.7	4.7	11.7	9.6
EPDM/Gel-Composite	46.9	0	23.4	3.7	4.7	11.7	9.6

**Table 3:** Mechanical properties of EPDM components with different fillers.

Samples	Tensile Strength (MPa)	Elongation at Break (%)
EPDM-0	0.94	431
EPDM-OPS	5.50	886
EPDM-Si	9.30	897
EPDM-Gel	9.03	1372

**Table 4:** Summary of the TGA results for silicon hybrid EPDM in air.

Samples	T_5%_ (°C)	T_max1_ (°C)	T_max2_ (°C)	Char Residue at 900 °C (%)	Theoretical Value Char Residue (%)
EPDM-0	329	409	512	1.9	--
EPDM-OPS	333	432	523	15.5	16.2
EPDM-Si	321	354	509	31.4	32.3
EPDM-Gel	317	419	508	27.3	28.3

**Table 5:** XPS data of EPDM-OPS, EPDM-Si, and EPDM-Gel.

Samples	C (at.%)	Si (at.%)	O (at.%)
EPDM-OPS	52.39	12.99	34.61
EPDM-Si	7.35	30.64	62.01
EPDM-Gel	5.00	30.54	64.45

**Table 6:** The back stable temperature and time of silicon hybrid EPDM at 500 °C or 1000 °C flame.

Samples	500 °C Flame		1000 °C Flame	
	Ts ^①^ (°C)	t-Ts ^②^ (s)	Ts ^①^ (°C)	t-Ts ^②^ (s)
EPDM-0	__	210 s burn-through	__	__
EPDM-OPS	__	1300 s burn-through	__	__
EPDM-Si	290~300	400~1500	__	110 s burn-through
EPDM-Gel	230~240	400~1500	__	117 s burn-through

① Ts: back stable temperature; ② t-Ts: time to reach the back stable temperature.

**Table 7:** TG and DTG data of silicon hybrid EPDM composites in air.

Code	T_5%_ (°C)	T_max1_ (°C)	R_max1_ (%/min)	T_max2_ (°C)	R_max2_ (%/min)	Char Residue at 900 °C (%)
EPDM-00	310.8	456.8	9.4	637.8	1.3	15.2
EPDM/Si-Composite	310.0	458.8	8.9	624.6	1.9	32.3
EPDM/Gel-Composite	276.3	456.8	8.1	606.2	1.7	27.7

**Table 8:** Back temperature data of silicon hybrid EPDM composites.

Code	500 °C Flame	1000 °C Flame at Different Times			
		100 s	300 s	600 s	800 s
EPDM-00	255~267 °C	144 °C	——	——	——
EPDM/Si-Composite	242~250 °C	172 °C	314 °C	361 °C	388 °C
EPDM/Gel-Composite	176~186 °C	118 °C	311 °C	369 °C	433 °C

**Table 9:** Pore parameters of the char skeleton of silicon hybrid EPDM composites at 1000 °C.

	Pore Volume (mL g^−1^)	Total Pore Area (m^2^ g^−1^)	Average Pore Diameter (nm)	Porosity (%)
EPDM-00	3.92	3.65	4297	83
EPDM/Si-Composite	3.10	115.04	108	76
EPDM/Gel-Composite	1.97	179.71	43	68

## Data Availability

Data are contained within the article and Supplementary Materials.

## Supplementary Materials

The following supporting information can be downloaded at: https://www.mdpi.com/article/10.3390/polym16050695/s1, Table S1. Principal characteristics of different fillers. Figure S1. (a). FTIR spectra of different fillers; (b). TGA-DTG curves of different fillers in air atmosphere; (c). XRD curve of different fillers.

## References

[1] Liu H., Zhu G., Zhang C. (2020). Promoted ablation resistance of polydimethylsiloxane via crosslinking with multi-ethoxy POSS. Compos. Part B Eng..

[2] Jelleab B.P. (2011). Air-filled nanopore based high-performance thermal insulation materials. Energy Build..

[3] Li C., Wu G., Li M., Hu C., Wei J. (2020). A heat transfer model for aluminum droplet/wall impact. Aerosp. Sci. Technol..

[4] Hao H., Zhou X., Shen Z., He J., Yang R. (2020). Study on the ablative properties of ethylene propylene diene terpolymer/silsesquioxane insulation materials. J. Appl. Polym. Sci..

[5] Rivier M., Lachaud J., Congedo P.M. (2019). Ablative thermal protection system under uncertainties including pyrolysis gas composition. Aerosp. Sci. Technol..

[6] Guo M., Li J., Xi K., Liu Y., Ji J. (2019). Effect of multi-walled carbon nanotubes on thermal stability and ablation properties of EPDM insulation materials for solid rocket motors. Acta Astronaut..

[7] Iqbal N., Sagar S., Khan M.B., Rafique H.M. (2014). Ablation and thermo-mechanical tailoring of EPDM rubber using carbon fibers. Polym. Eng. Sci..

[8] Gao G., Zhang Z., Li X., Meng Q., Zheng Y. (2010). An excellent ablative composite based on PBO reinforced EPDM. Polym. Bull..

[9] Natali M., Rallini M., Kenny J., Torre L. (2016). Effect of Wollastonite on the ablation resistance of EPDM based elastomeric heat shielding materials for solid rocket motors. Polym. Degrad. Stab..

[10] Natali M., Monti M., Kenny J.M., Torre L. (2011). A nanostructured ablative bulk molding compound: Development and characterization. Compos. Part A Appl. Sci. Manuf..

[11] Singh S., Guchhait P.K., Bandyopadhyay G.G., Chaki T.K. (2013). Development of polyimide–nanosilica filled EPDM based light rocket motor insulator compound: Influence of polyimide–nanosilica loading on thermal, ablation, and mechanical properties. Compos. Part A Appl. Sci. Manuf..

[12] Khan H., Amin M., Yasin M., Ali M., Ahmad A. (2016). Structure and properties of particles/rubber composites applied on functionally graded lapping and polishing plate. J. Polym. Eng..

[13] Morselli D., Bondioli F., Luyt A.S., Mokhothu T.H., Messori M. (2013). Preparation and characterization of EPDM rubber modified with in situ generated silica. J. Appl. Polym. Sci..

[14] Jiang X., Zhao Z., Zhou S., Zou H., Liu P. (2022). Anisotropic and lightweight carbon/graphene composite aerogels for efficient thermal insulation and electromagnetic interference shielding. Appl. Mater. Interfaces.

[15] Shi S., Lei B., Li M., Cui X., Wang X., Fan X., Tang S., Shen J. (2020). Thermal decomposition behavior of a thermal protection coating composite with silicone rubber: Experiment and modeling. Prog. Org. Coat. Int. Rev. J..

[16] Jin R., Zhou Z., Liu J., Shi B., Zhou N., Wang X., Jia X., Guo D., Xu B. (2023). Aerogels for Thermal Protection and Their Application in Aerospace. Gels.

[17] Serge B., Johan S., Tsilla B. (2020). Intumescent polypropylene in extreme fire conditions. Fire Saf. J..

[18] (2009). Determination of Tensile Stress-Strain Properties of Vulcanized or Thermoplastic Rubber.

[19] Sohrab A., Gelareh M., Claudiane O.-P., Eric D. (2020). Performance improvement of EPDM and EPDM/Silicone rubber composites using modified fumed silica, titanium dioxide and graphene additives. Polym. Test..

[20] Lee S.-Y., Singh P., Mahajan R.L. (2019). Role of oxygen functional groups for improved performance of graphene-silicone composites as a thermal interface material. Carbon.

[21] Xue B., Yang S., Qin R., Deng S., Niu M., Zhang L. (2022). Effect of a graphene-APP composite aerogel coating on the polyester fabric for outstanding flammability. Prog. Org. Coat..

