# Supplementary material for: Silicon Hybrid EPDM Composite with High Thermal Protection Performance
Source: Polymers (Basel). 2024 Mar 4;16(5):695. doi: 10.3390/polym16050695 (PMC10933853; doi:10.3390/polym16050695)
Supplement: Supplementary file 1 [file polymers-16-00695-s001.zip › polymers-2888021-supplementary.pdf]

Supplementary Information

Silicon hybrid EPDM composite with high thermal protection performance

Chenyang Yan [1], Bo Chen [1], Xiangmei Li [1*], Jiyu He [1*], Xin Zhao[2],Yanli Zhu[3], Rongjie Yang [1]

1 National Engineering Research Center for Flame Retardant Materials, School of Materials science and engineering, Beijing Institute of Technology, Beijing 100081, China
2 Beijing Chemical Industry Research Institute.Co.,Ltd.,Beijing 100080,China
3 School of Mechatronical Engineering, Beijing Institute of Technology, Beijing 100081, China

Molecular structure

Table S1 Principal characteristics of different fillers

Material	state	Specific surface area （m^2/g）	Total pore volume （ml/g）
Silica aerogel	white powder	621	3.09
Fumed silica	white powder	190	0.64
OPS	white powder	2.69	0.007

The FTIR spectra of OPS、silica and aerogel are shown in Fig .1-a, where both the silica and aerogel have two strong absorption peaks around 3440cm^{-1} and 1640cm^{-1} assigned to the O-H stretching vibration of hydrogen bonds in water molecules and the anti-symmetric stretching vibration peak of Si-O-Si respectively, which proves the existence of SiO_2 network structure. The absorption peak of OPS at 697 cm^{-1} and 747 cm^{-1} is the out-of-plane bending vibration absorption peak of hydrogen on the single substituted benzene ring. The absorption peak at 1130 cm^{-1} and 1430 cm^{-1} is the Si-Ph stretching vibration peak, and the C-C stretching vibration absorption peak on the benzene ring in Si-Ph is at 1600 cm^{-1}.

The TGA and DTG patterns of OPS、silica and aerogel carried out in air are reported in Fig. 1-b .At 800°C, the weight loss curves of Silica and Aerogel are relatively gentle, and the residues of both are higher than that of OPS,which is only

* corresponding author: bjlglxm@bit.edu.cn
* corresponding author: hejiyu@bit.edu.cn

one weight-loss stage in the TG curve of OPS, and $T_{5\%}$ and T_{max} correspond to 457.1℃ and 530.2℃,at 800℃, the residual amount of 46.9% is still there. The weight loss of OPS in the temperature range of 478-554℃ is mainly related to the degradation of peripheral organic groups in the molecular structure of OPS, and the decomposition of Si-Si-O cage structure to form SiO_2,SiOxCy and SiC at higher temperatures， which corresponds to the molecular structure in table 1.

XRD of OPS shows an obvious crystal structure peak, which is closely related to its highly symmetrical three-dimensional structure. As shown in Fig.1-c, the OPS angles at 8.11˚,8.35 ˚, 18.48˚,19.38˚ and 24.87˚ are on the 100,010,110,001,120 and 022 planes, respectively. The peak at the wide Angle of 8.11˚ is the Si-O cage structure peak, while the Silica and Aerogel show obvious amorphous structure.

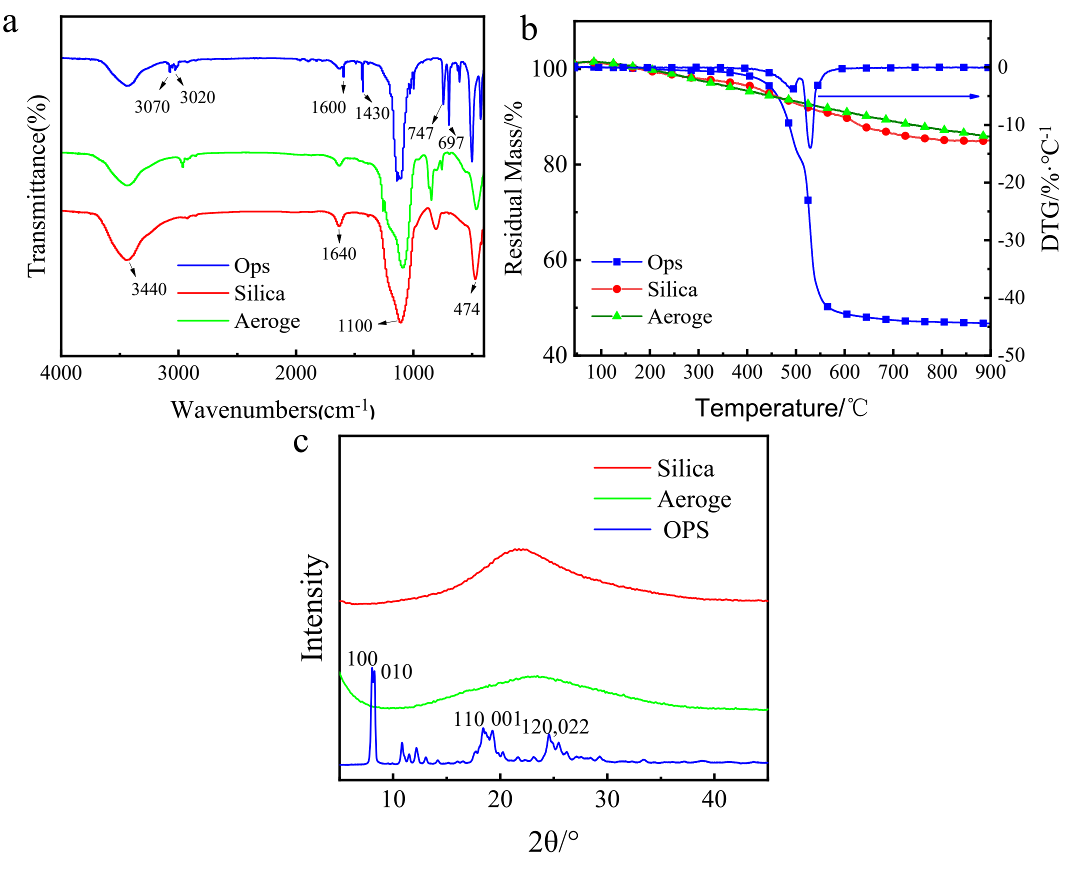

Figure S1. (a).FTIR spectra of different fillers.

(b).TGA-DTG curves of different fillers in air atmosphere

(c). XRD curve of different fillers
